# Comparing Supervised Exercise Therapy to Invasive Measures in the Management of Symptomatic Peripheral Arterial Disease

**DOI:** 10.1155/2015/960402

**Published:** 2015-10-27

**Authors:** Thomas Aherne, Seamus McHugh, Elrasheid A. Kheirelseid, Michael J. Lee, Noel McCaffrey, Daragh Moneley, Austin L. Leahy, Peter Naughton

**Affiliations:** ^1^Department of Vascular Surgery, Beaumont Hospital, Dublin 9, Ireland; ^2^Department of Interventional Radiology, Beaumont Hospital, Dublin 9, Ireland; ^3^Department of Human and Health Performance, Dublin City University, Dublin 9, Ireland

## Abstract

Peripheral arterial disease (PAD) is associated with considerable morbidity and mortality. Consensus rightly demands the incorporation of supervised exercise training (SET) into PAD treatment protocols. However, the exact role of SET particularly its relationship with intervention requires further clarification. While supervised exercise is undoubtedly an excellent tool in the conservative management of mild PAD its use in more advanced disease as an adjunct to open or endovascular intervention is not clearly defined. Indeed its use in isolation in this cohort is incompletely reported. The aim of this review is to clarify the exact role of SET in the management of symptomatic PAD and in particular to assess its role in comparison with or as an adjunct to invasive intervention. A systematic literature search revealed a total 11 randomised studies inclusive of 969 patients. All studies compared SET and intervention with monotherapy. Study results suggest that exercise is a complication-free treatment. Furthermore, it appears to offer significant improvements in patients walk distances with a combination of both SET and intervention offering a superior walking outcome to monotherapy in those requiring invasive measures.

## 1. Introduction

Peripheral arterial disease (PAD) affects 12–16% of the population over the age of 60 years with intermittent claudication (IC), its primary symptom, proving detrimental to patient quality of life [1–4]. Typically PAD follows a stable course with management confined to conservative measures; however one in ten PAD patients will develop critical limb ischaemia (CLI) with all-cause mortality in the CLI cohort rising to 50% at 5 years [5–7]. This reduction in life expectancy is due largely to concomitant cardiovascular disease [8, 9].

Therefore, treatment goals should focus not only on the alteration of disease progression and symptomatic relief but also on the improvement of patient long-term survival [10]. Approaches include the modification of risk factors through optimum medical therapy (OMT) and supervised exercise therapy (SET) with endovascular (EVR) and open surgical revascularization reserved for those failing conservative measures. Further novel therapies including kinesitherapy and electrotherapeutic procedures have also been proposed [11]. While endovascular treatment offers a minimally invasive revascularization option for many patients data supporting its ability to improve long-term survival is lacking. Regular exercise, on the other hand, is associated with a 50% reduction in cardiovascular mortality [12]. Supervised exercise training consists of a prescribed, evidence based exercise program which is performed under the direct observation of a trained practitioner. It is now well established as an initial noninvasive option in all PAD patients with robust supporting data [13–15].

*Rationale for Review*. Despite its intuitive benefits a wide variation in the use and availability of SET exists and it remains a greatly underutilized resource due to limited patient access [16]. The BASIL study highlights the deficiencies in the current medical optimization with few participants utilizing clinically proven best medical therapy [7, 17]. The issue is further clouded by conflicting literature as to the optimal nonsurgical management of these patients [18–32]. Thus while its use is strongly supported by current literature it appears that SET is underutilized in mild-to-moderate PAD while its use in more advanced disease requires further clarification. With this likely in mind the Institute of Medicine has prioritized research into the comparative efficacy of the different treatment modalities for PAD [33]. Furthermore, the role of SET as an adjunct to or substitute for intervention remains unclear.

The aim of this review was to compare the use of supervised exercise therapy to invasive measures in the management of symptomatic peripheral arterial disease thus clarifying an exact role for SET in the management of this patient cohort.

## 2. Methods

### 2.1. Study Eligibility

All randomised controlled trials (RT) assessing exercise in conjunction with or in comparison to an endovascular or open intervention in the management of peripheral arterial disease were included for review (Table 1). All observational and review data were excluded from the results. Relevant papers were searched and evaluated independently by two assessors. Outcomes were tabulated where figures were included.

### 2.2. Literature Search

The online medical literature database PUBMED was systematically searched. All studies and relevant reviews were manually cross-referenced to identify any outstanding articles.

**Figure 1:**
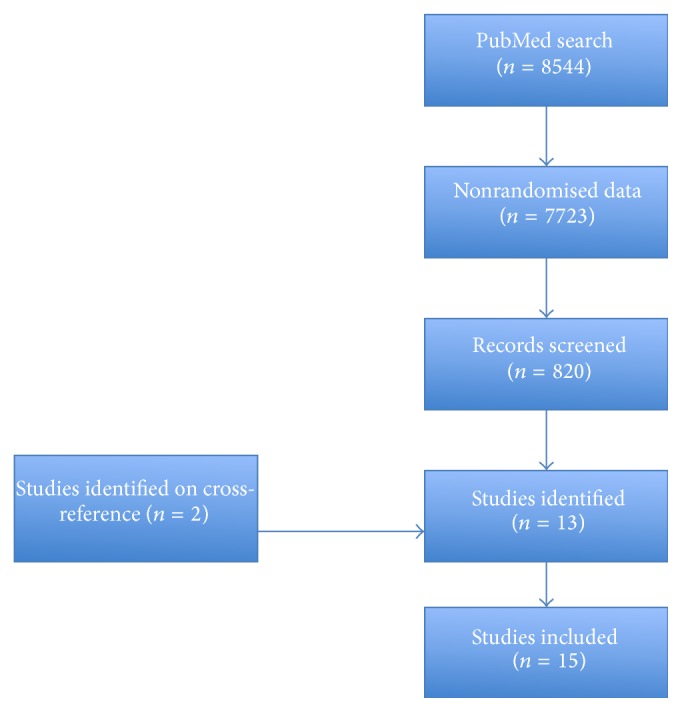
Flow diagram depicting study identification.

PubMed was last searched on September 18, 2015 (Figure 1). The database was comprehensively searched without date or language restriction using the following search strategy.

[[[[[[[[peripheral arterial disease] OR peripheral vascular disease] OR claudication] AND angioplasty] OR revascularization] OR endovascular] OR open surgery] OR bypass] AND exercise. A total of 8544 studies were identified. After the filter for randomised controlled trials was applied 820 studies were identified. Relevant full articles were reviewed by two reviewers [TA, PN].

## 3. Results

A total of 15 papers (Table 1) report outcomes of 11 RT. These trials include a total of 969 patients and all directly compare supervised exercise with various invasive interventions. Maximum walking distance (MWD), intermittent claudication distance (ICD), and ankle brachial pressure index (ABPI) measurement form the cornerstones of vascular assessment in each study.

### 3.1. Quality Assessment of Assessed Data

The risk of bias in each included study is summarised in Table 2. Few papers reported any participant heterogeneity with regard to baseline function, comorbidities, and smoking status. Risk assessment was performed with guidance from the Cochrane Handbook for Systematic Reviews of Interventions [34].

### 3.2. Supervised Exercise versus Endovascular Intervention

Five trials including 519 patients directly compare the outcomes of EVR and SET in the management of peripheral arterial disease (Table 3).

At six months Murphy et al. reported significant improvements in maximum walk times in those undergoing SET compared to those in the EVR group [18]. However, at 18-month follow-up this benefit was lost with no significant difference in walk times identified [32]. ABPI were consistently higher in the EVR group. Creasy and Perkins noted significant improvements in the functionality of both groups [19, 20]. Again, no significant change in ABPI was noted in any SET cohort. Improvement in mobility was most significant when the disease affected the superficial femoral artery. Hobbs et al. only noted significant improvements in walk distances in those receiving EVR [21]. No improvement was seen with SET alone. Spronk et al. reported a 1-week clinical success rate of 88% following EVR decreasing to 68% at 12 months while the SET group had an early success rate of 16% increasing to 65% by 12 months [22, 23]. Clinical success was defined as an improvement in at least one category in the Rutherford scale posttreatment. Long-term outcomes (7 years) from this RT show maintenance of the functional gains achieved at 1 year with no variance in amputation rate 10 between groups [24]. Finally, Mazari et al. identified significant functional improvements in both groups at one year; however no statistically significant difference was identified between cohorts [25]. Again, only EVR was associated with improved ABPI measurements.

### 3.3. Supervised Exercise Plus Invasive Measures (Open Surgery or EVR) versus Monotherapy

In total six studies inclusive of 514 patients compare the merits of combination therapy consisting of invasive intervention and SET and single intervention alone (Table 4). Two included studies examine the benefits of a combination of open arterial surgical reconstruction and SET [30, 31] while four articles assess dual therapy including both EVR and SET [25–28].

The earliest work in this area by Lundgren et al. compares open arterial reconstruction and SET with open surgery alone [30]. This study identified improvements in walk distances in both groups; however, those undergoing combination therapy experienced significantly improved walking performance at 13 months. Similarly, Badger et al. identified significant improvements in MWD in patients undergoing peripheral arterial bypass in conjunction with SET compared to those undergoing bypass in isolation [31].

More recently, studies have focused on the use of SET as an adjunct to EVR. Mazari et al. identified that a combined treatment group achieved the greatest (but not statistically significant) improvement in MWD and ICD with a lower incidence of reintervention compared to the monotherapy groups [25]. This benefit was further supported by randomised data from Greenhalgh et al. [26–28]. At six months Kruidenier et al. identified significantly lengthened walk distances when SET was used as an adjunct to EVR. Furthermore, both Greenhalgh and Bø et al. examined patients with both aortoiliac and infrainguinal disease in separate trial limbs. While Greenhalgh compared combination therapy with SET alone Bø et al. contrasted dual therapy with EVR alone. Both studies identified improvements in walk distances in both trial limbs for patients undergoing combination therapy versus monotherapy; however only Greenhalgh identified significantly better ABPI in the dual intervention group.

### 3.4. Invasive (EVR or Surgery) Management versus Supervised Exercise

Gelin alone compared invasive intervention (either EVR or open surgery based on preoperative angiography) and supervised exercise [29] (Table 5). At one year only those randomised to invasive measures experienced any improvement in walk distance or lower limb arterial pressures.

### 3.5. Open Surgery versus Supervised Exercise

Lundgren et al. included a comparison of open revascularisation and SET in the previously discussed RT (Table 4) [30]. At 13 months those undergoing open surgery had better functional performance than those undergoing SET alone. In addition, those undergoing surgery experienced a significantly higher ABPI to those in the exercise group.

## 4. Discussion

Peripheral arterial disease is a widespread phenomenon in the elderly population [35]. The optimum management of claudication continues to raise debate. Exercise is a straightforward and effective conservative treatment option. Meta-analysis has shown a 122% increase in walking distances in patients undergoing exercise therapy [15]. This has been reinforced by the more recent Cochrane review examining exercise for IC [36]. However, despite its success intensive programs continue to be associated with high dropout rates [37].

The exact physiological mechanism by which exercise improves performance is incompletely understood. Multiple physiological adaptions have been proposed as contributing factors. Arterial collateralization has the potential to improve peripheral blood flow in the ischaemic limb with the exercised muscle displaying increased levels of the proangiogenic vascular endothelial growth factor [38]. However, improved functional performance in the trained limb is not reflective of improved ABPI measurements as seen in endovascular revascularization [18, 39]. Increased arterial shear stress in exercise is associated with nitric oxide (NO) release, a powerful vasoactive agent [40]. This endothelial-mediated response is impaired in patients with PAD [41]. The concept of “hemorheologic fitness” suggests that a proven reduction in blood viscosity in the trained individual may result in improved peripheral metabolic efficiency [42]. In addition, gait proficiency, reversal of acquired metabolic myopathies, and modified inflammatory responses all have the potential to improve exercise related function [43–46].

**Figure 2:**
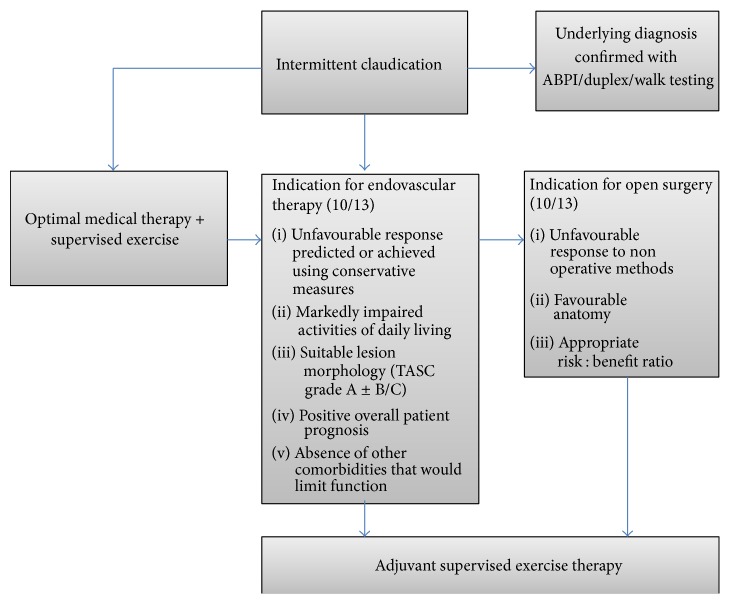
Management of intermittent claudication incorporating supervised exercise therapy.

This review suggests a number of roles for supervised exercise therapy in the symptomatic PAD patient (Figure 2). Significantly, no study reported exercise related complications.

- Direct comparison of SET and endovascular measures revealed similar functional outcomes for both interventions in the medium term across a number of studies with long-term data from one study identifying comparable limb salvage between groups. However, only EVR alone resulted in significant improvements in lower limb perfusion as measured by ABPI. These data have significant applicability to symptomatic patients whose comorbidities allow them to exercise to an adequate level for SET. Supervised exercise offers this group an acceptable, effective initial step in those capable and motivated to take part.
- In those requiring intervention a combination of surgical intervention and SET offered superior outcomes to monotherapy across multiple studies. Two trials assessed open surgery with adjunctive SET with both suggesting significant benefits in walk distances in the dual therapy group. Similarly, all four trials assessing endovascular intervention in conjunction with SET compared to monotherapy found greater improvements in the walking distances of those undergoing dual therapy. These data would strongly support the use of SET as an adjunct to any operative intervention in the management of symptomatic PAD.
- Only one study directly compared open surgery and SET. This strongly supported the surgical approach in terms of both function and perfusion outcomes.

Multiple exercise modalities including treadmill walking, resistance training, and upper limb ergometry have proven benefit in PAD [12, 36, 47, 48]. Unfortunately, the success of SET is reliant on excellent compliance and availability. Supervised exercise training has been shown to consistently result in improved functionality with superior outcomes to a “go home and walk” approach [49–51]. Compliance can further be augmented by regular exercise reminders, patient specific exercise prescriptions, target setting, and the incorporation of exercise into daily activities [52]. Gains in walk time and claudication onset occur rapidly over the initial 2 months and can be maintained with good compliance [53]. An exercise prescription of at least two 30-minute sessions per week offers optimal walking outcomes [54].

In those failing or unable to partake in an initial exercise program endovascular revascularization offers low rates of periprocedural morbidity and mortality with excellent initial success rates [55–57]. However the two main shortcomings of endovascular intervention include long-term durability and overall poor survival rates associated with concomitant cardiovascular disease. Supervised exercise addresses some of these concerns. By stimulating collateralization and the release of vasodilators such as nitric oxide exercise therapy should confer superior functional results in the longer-term. This is supported by a number of studies [58, 59]. Thus, for this patient group SET offers not only functional improvement but also significant benefits to the cardiovascular health of these patients [12].

### 4.1. Strengths and Limitations

This review is strengthened by the fact that all incorporated data are from well-designed randomised controlled studies. Follow-up ranged from 6 months to 12 years. Cumulatively these trials incorporate a significant number of claudicants. Individually papers provide strong data that exercise results in significant gains in walk distance. In addition, a number of studies have found that a combination of SET and intervention provides a superior outcome to monotherapy.

This review is however subject to a number of limitations. Firstly, while all patients had significant arterial symptoms a degree of heterogeneity exists in this cohort. Each included study describes slight variations in the level of disease, the symptomatic presentation, and the intervention received rendering definitive conclusion difficult. Secondly, many studies included small numbers of participants with a number reporting moderate losses to follow-up. Finally, due to the heterogeneity of outcome data meta-analysis was not performed as part of this review.

## 5. Conclusion

Exercise is an effective, safe, and economical method of treating symptomatic PAD [23]. Some randomised data now suggests that it may be comparable to EVR in the management of symptomatic noncritical ischaemia. Furthermore there is emerging evidence that combining endovascular intervention following by supervised exercise achieves better long-term results than endovascular intervention alone; however greater surgeon access to and awareness of this important adjuvant therapy is essential to maximize therapeutic outcomes.

## Figures and Tables

**Table 1:** Treatment groups and disease level.

Study	Patient number	Intervention						Disease level		
		EVR	SET	EVR + SET	INV	Surgery	Surgery + SET	Fem-pop	Aortoiliac	Multilevel
Lundgren et al. [30]	75		25			25	25	Not recorded		
Creasy et al./Perkins et al. [19, 20]	56	30	26					28	25	0
Gelin et al. [29]	164		88		66			Not recorded		
Hobbs et al. [21]	23	9	7					23	0	0
Badger et al. [31]	14		6				8	14	0	0
Greenhalgh et al. [26]	127		60	67				93	94	
Kruidenier et al. [27]	70	35		35				5	60	5
Mazari et al. [25]	178	60	60	58				Not recorded		
Spronk et al./Fakhry et al. [22–24]	151	76	75					44	106	—
Murphy et al. [18, 32]	111	46	43					0	111	0
Bø et al. [28]	50	21		29				25	25	0

EVR: endovascular revascularization; SET: supervised exercise therapy; INV: invasive management; Fem-pop: femoropopliteal.

**Table 2:** Assessment of bias.

Lundgren et al. [30]	Random sequence generation	Randomised but not described	Unclear risk of bias
	Allocation concealment	Randomised but not described	Unclear risk of bias
	Blinding of participants and personnel	Blinding not possible	Not assessed
	Incomplete outcome data	Follow-up data in each group incomplete	Moderate risk of bias
	Selective reporting	Clear outcomes	Low risk of bias
	Other sources of bias	None	Low risk of bias

Creasy et al./Perkins et al. [19, 20]	Random sequence generation	Randomised but not described	Unclear risk of bias
	Allocation concealment	Randomised but not described	Unclear risk of bias
	Blinding of participants and personnel	Blinding not possible	Not assessed
	Incomplete outcome data	No loss to follow-up reported	Low risk of bias
	Selective reporting	Clear outcomes	Low risk of bias
	Other sources of bias	None	Low risk of bias

Gelin et al. [29]	Random sequence generation	Randomised via computer based algorithm	Low risk of bias
	Allocation concealment	Randomised via computer based system	Low risk of bias
	Blinding of participants and personnel	Blinding not possible	Not assessed
	Incomplete outcome data	Some loss to follow-up	Moderate risk of bias
	Selective reporting	Clear outcomes	Low risk of bias
	Other sources of bias	None	Low risk of bias

Hobbs et al. [21]	Random sequence generation	Randomised with 2 × 2 factorial design	Low risk of bias
	Allocation concealment	Computer generated randomisation	Low risk of bias
	Blinding of participants and personnel	Blinding not possible	Not assessed
	Incomplete outcome data	Four withdrawals	Low risk of bias
	Selective reporting	Clear outcomes	Low risk of bias
	Other sources of bias	None	Low risk of bias

Badger et al. [31]	Random sequence generation	Randomised but not described	Unclear risk of bias
	Allocation concealment	Randomised but not described	Unclear risk of bias
	Blinding of participants and personnel	Blinding not possible	Not assessed
	Incomplete outcome data	All patients lost to 6-month follow-up	High risk of bias
	Selective reporting	Clear outcomes	Low risk of bias
	Other sources of bias	None	Low risk of bias

Greenhalgh et al. [26]	Random sequence generation	Detailed description of Stata generated randomisation	Low risk of bias
	Allocation concealment	Computer generated randomisation	Low risk of bias
	Blinding of participants and personnel	Blinding not possible	Not assessed
	Incomplete outcome data	Moderate loss to follow-up	Moderate risk of bias
	Selective reporting	Clear outcomes	Low risk of bias
	Other sources of bias	None	Low risk of bias

Kruidenier et al. [27]	Random sequence generation	Computer generated block randomisation	Low risk of bias
	Allocation concealment	Computer generated block randomisation	Low risk of bias
	Blinding of participants and personnel	No blinding	High risk of bias
	Incomplete outcome data	Moderate losses to follow-up	Moderate risk of bias
	Selective reporting	Clear outcomes	Low risk of bias
	Other sources of bias	None	Low risk of bias

Mazari et al. [25]	Random sequence generation	Sealed envelope used to randomise	Low risk of bias
	Allocation concealment	Sealed envelope used to randomise	Low risk of bias
	Blinding of participants and personnel	Blinding not described	Unclear risk of bias
	Incomplete outcome data	Moderate loss to follow-up	Moderate risk of bias
	Selective reporting	Clear outcomes	Low risk of bias
	Other sources of bias	None	Low risk of bias

Spronk et al./Fakhry et al. [22–24]	Random sequence generation	Computer generated block randomisation	Low risk of bias
	Allocation concealment	Computer generated block randomisation	Low risk of bias
	Blinding of participants and personnel	Blinding not possible	Not assessed
	Incomplete outcome data	Prolonged study with some loss to follow-up	Moderate risk of bias
	Selective reporting	Clear outcomes	Low risk of bias
	Other sources of bias	None	Low risk of bias

Murphy et al. [18, 32]	Random sequence generation	Web based randomisation	Low risk of bias
	Allocation concealment	Web based randomisation	Low risk of bias
	Blinding of participants and personnel	Observers blinded	Low risk of bias
	Incomplete outcome data	Prolonged study with some loss to follow-up	Moderate risk of bias
	Selective reporting	Clear outcomes	Low risk of bias
	Other sources of bias	None	Low risk of bias

Bø et al. [28]	Random sequence generation	Computer based randomisation	Low risk of bias
	Allocation concealment	Computer based randomisation	Low risk of bias
	Blinding of participants and personnel	Observers blinded	Low risk of bias
	Incomplete outcome data	No loss to follow-up	Low risk of bias
	Selective reporting	Clear outcomes	Low risk of bias
	Other sources of bias	None	Low risk of bias

**Table 3:** Supervised exercise versus endovascular revascularization.

Study	Follow-up	ABPI		*P*	MWD		*P*	ICD		*P*
		Baseline	Endpoint		Baseline	Endpoint		Baseline	Endpoint	
Hobbs et al. [21]										
EVR	6 months	0.69	0.93	0.013^†^	185	698	0.008^†^	84	698	0.011^†^
SET		0.66	0.70	0.46^†^	111	124	0.35^†^	59	92	0.074^†^
Mazari et al. [25]										
EVR	12 months	0.71	0.90		77	146		31	75	
SET		0.72	0.84	0.093	83	215	0.2	42	97	0.48
Spronk et al./Fakhry et al. [22–24]										
EVR	7 years	0.63	0.84		174	1248		82	1022	
SET		0.62	0.82	0.8	186	1168	0.48	104	804	0.15
Murphy et al. [18, 32]										
EVR	18 months	0.7	0.7		5.2 min	8.4 min		1.8 min	4.8 min	
SET		0.6	0.6	<0.001	5.6 min	10.6 min	0.16	1.8 min	5.1 min	0.77

ABPI: ankle brachial pressure index; MWD: maximal walk distance; ICD: intermittent claudication distance; EVR: endovascular revascularization; SET: supervised exercise therapy. Distances in metres unless otherwise stated. min represents time in minutes, claudication onset time/maximal walk time. *P* value represents statistical comparison of interventions except in Hobbs et al. [21] where † represents change in measurement over study period.

**Table 4:** Supervised exercise plus invasive measures (open surgery or EVR) versus monotherapy.

Study	Follow-up	ABPI		*P*	MWD		*P*	ICD		*P*
		Baseline	Endpoint		Baseline	Endpoint		Baseline	Endpoint	
Lundgren et al. [30]										
Surgery	13 months	0.55	—		209	570		85	405	
SET		0.59	—		180	654		70	559	
Surgery + SET		0.59	—	<0.001	183	459	0.05	67	187	0.006
Badger et al. [31]										
Surgery	6 months	—	—		—	—		—	—	—
Surgery + SET		—	—	0.02^*∗*^	—	—	0.001^*∗*^	—	—	—
Greenhalgh et al. [26] (Aortoiliac)										
SET	24 months	0.66	0.74		126	168		—	—	—
EVR + SET		0.68	0.90	0.02	114	354	0.05	—	—	—
Greenhalgh et al. [26] (Femoropopliteal)										
SET	24 months	0.69	0.72		126	155		—	—	—
EVR + SET		0.66	0.83	0.01	133	245	0.4	—	—	—
Kruidenier et al. [27]										
EVR	6 months	0.71	0.93		282	685		343	547	
EVR + SET		0.69	0.88	0.755	186	956	0.001	293	842	0.001
Mazari et al. [25]										
EVR	12 months	0.71	0.9		77	146		31	75	
SET		0.72	0.84		83	215		42	97	
EVR + SET		0.64	0.92	0.93	85	187	0.259	43	99	0.484
Bø et al. [28]										
EVR	3 months	—	—		213	427		94	267	
EVR + SET		—	—	<0.001^*∗*^	385	584	NS	101	456	NS

ABPI: ankle brachial pressure index; MWD: maximal walk distance; ICD: intermittent claudication distance; EVR: endovascular revascularization; SET: supervised exercise therapy. Distances in metres unless otherwise stated. *∗* represents significant improvement favouring combined treatment. *P* value represents statistical comparison of interventions. — represents nonreported figures.

**Table 5:** Invasive (EVR or surgery) management versus supervised exercise.

Study	Follow-up	ABPI		*P*	MWD		*P*	ICD		*P*
		Baseline	Endpoint		Baseline	Endpoint		Baseline	Endpoint	
Gelin et al. [29]										
INV	12 months	0.55	0.71	<0.01^†^	274	344	<0.01^†^	—	—	—
SET		0.56	0.54	—	258	247	—	—	—	—

ABPI: ankle brachial pressure index; MWD: maximal walk distance; ICD: intermittent claudication distance; EVR: endovascular revascularization; SET: supervised exercise therapy. Distances in metres unless otherwise stated. *P* value represents statistical comparison of interventions. † represents change in measurement over study period.
